# Stepwise Identification Method of Thermal Load for Box Structure Based on Deep Learning

**DOI:** 10.3390/ma17020357

**Published:** 2024-01-10

**Authors:** Hongze Du, Qi Xu, Lizhe Jiang, Yufeng Bu, Wenbo Li, Jun Yan

**Affiliations:** 1 State Key Laboratory of Structural Analysis for Industrial Equipment, School of Mechanics and Aerospace Engineering, Dalian University of Technology, Dalian 116024, China; duhongze@mail.dlut.edu.cn (H.D.); jiang521@mail.dlut.edu.cn (L.J.); buyufeng@mail.dlut.edu.cn (Y.B.); liwb14@163.com (W.L.); 2 Ningbo Research Institute, Dalian University of Technology, Ningbo 315016, China; namexuqi@163.com

**Keywords:** thermal load identification, stepwise identification method, deep learning, boundary condition encoding

## Abstract

Accurate and rapid thermal load identification based on limited measurement points is crucial for spacecraft on-orbit monitoring. This study proposes a stepwise identification method based on deep learning for identifying structural thermal loads that efficiently map the local responses and overall thermal load of a box structure. To determine the location and magnitude of the thermal load accurately, the proposed method segments a structure into several subregions and applies a cascade of deep learning models to gradually reduce the solution domain. The generalization ability of the model is significantly enhanced by the inclusion of boundary conditions in the deep learning models. In this study, a large simulated dataset was generated by varying the load application position and intensity for each sample. The input variables encompass a small set of structural displacements, while the outputs include parameters related to the thermal load, such as the position and magnitude of the load. Ablation experiments are conducted to validate the effectiveness of this approach. The results show that this method reduces the identification error of the thermal load parameters by more than 45% compared with a single deep learning network. The proposed method holds promise for optimizing the design and analysis of spacecraft structures, contributing to improved performance and reliability in future space missions.

## 1. Introduction

As the most common structural form in spacecrafts, the box-type structure, accurate real-time acquisition of its thermal environment is meaningful to intelligently assessing the service status of on-orbit structures and is crucial in equipment health monitoring and fault diagnosis [1]. Additionally, real-time acquisition of structural thermal load information can assist engineers in offline maintenance and online feedback control [2,3] as well as applicable to aerospace engineering [4,5,6], flight vehicles [7], high-performance machining [8], and thermal experimental device development [9]. However, one may not be able to directly measure the thermal loads on a structure owing to the severity of the environment or the inaccessibility of heat sources [10], which implies that only a few local responses can be obtained. The precise thermal load exerting on the structure must be determined despite the limited local response data. 

Thermal load identification is an inverse problem that differs significantly from other forward structural computations. When the local responses reach the overall load in the inverse analysis, slight random errors in the data are magnified, thus resulting in significant inaccuracies and unsatisfactory conditioning [6,10]. This phenomenon is of interest to many researchers because it is challenging and useful in practical applications. For materials with simple and regular shapes, precise solutions can be obtained by solving inverse heat conduction [7,11,12] and inverse thermoelasticity problems [10,13,14]. However, as the geometrical complexity of the structures increases, the solution process necessitates lengthy and complicated mathematical derivations, extensive numerical computations, and high-quality response measurements [15]. Real-time and accurate thermal load identification of complex structures remains a formidable challenge in engineering applications.

Owing to their ease of implementation and rapid online computation, machine learning algorithms offer a new approach for rapidly identifying complex structural loads by directly uncovering the underlying relationship between responses and loads from data. Academics began investigating load identification based on machine learning as early as the 1990s [16], where they employed artificial neural networks to model the strain–load relationship of structures and perform load identification. Subsequently, other machine learning algorithms, such as Monte Carlo simulation methods [17], random forests [18], Elman networks [19], and backpropagation neural networks [20,21,22,23] have been shown to successfully identify loads. However, classical machine learning algorithms tend to manage individual tasks separately, thus rendering it challenging to satisfy the accuracy requirements of tasks involving the simultaneous identification of load locations and magnitudes.

Deep learning algorithms allow the designs of several neural network modules to be combined, thus enabling the deep abstraction of input features and providing greater nonlinear analytical capabilities for addressing more challenging load identification problems. Ren et al. [24] proposed a deep learning algorithm that directly determines the structural damage load parameters from residual plastic deformations. Wada et al. [25] achieved the recognition of the complex distributed static load by performing shape function interpolation on the output nodes gained by neural networks. Feng et al. [26] utilized convolutional neural networks (CNNs) to automatically extract features from the time-frequency signals of guided wave data, thereby achieving low-velocity impact load localization for structures. Yang et al. [27] discovered that convolutional layers can be regarded as filters in dynamic load identification and that their proposed deep CNN exhibited high resistance to noise. In other studies, recurrent neural networks (RNNs) for load detection were investigated [28], and the results showed that the RNN algorithm was not affected significantly by sensor arrangement and measurement errors [29]. Du et al. [30] employed the full-field temperature gradient and temperature variation rate of honeycomb sandwich structures under laser irradiation as input parameters for a deep learning model. They established a loss function guided by physical information through the introduction of a laser power density function, enabling high-precision identification of thermal load parameters (laser diameter and power). Deep learning algorithms have been demonstrated to be advantageous over conventional machine learning approaches. In addition, distributing a challenging load identification task to several deep learning models for regressing load parameters has been shown to yield high accuracy. However, in the aforementioned studies, the interrelationships between the load parameters and the geometric boundary characteristics of the structure were not considered. In fact, each deep learning model was individually trained and tested, which reduced the efficiency of the method construction and generalization performance on different structures. Furthermore, to the best of our knowledge, the application of a deep learning approach for identifying thermal loads has not been reported. Thus, this study was conducted to address this gap.

Herein, a stepwise identification method based on deep learning for the inverse identification of structural thermal loads is proposed, which includes the localization of the load position and the estimation of its magnitude. Prior knowledge regarding the boundary conditions (BCs) is incorporated into a cascaded deep learning model to gradually achieve more accurate thermal load identification while considering the geometric boundary characteristics of various regions and the relationships among the different thermal load parameters.

## 2. Method: Stepwise Identification Method Based on Deep Learning

### 2.1. Method Description

For a structural instance and response data $U\in {\mathbb{R}}^{n}$ measured from n locations, the overall thermal load Q(x,q) exerting on the structure must be determined, including the position $x\in {\mathbb{R}}^{d}$ and magnitude $q\in {\mathbb{R}}^{1}$ of the load, where x and q are a d-dimensional vector (d is the geometric dimension of the structure) and scalar, respectively. Determining the inverse relationship $F^{-1}\left(\cdot \right)$ between U and Q and then entering the response data to identify the global load $Q=F^{-1}\left(U\right)$ is the primary goal of load identification. Therefore, the accuracy and efficiency of load identification significantly depend on the inverse relationship model $F^{-1}\left(\cdot \right)$.

**Figure 1:**
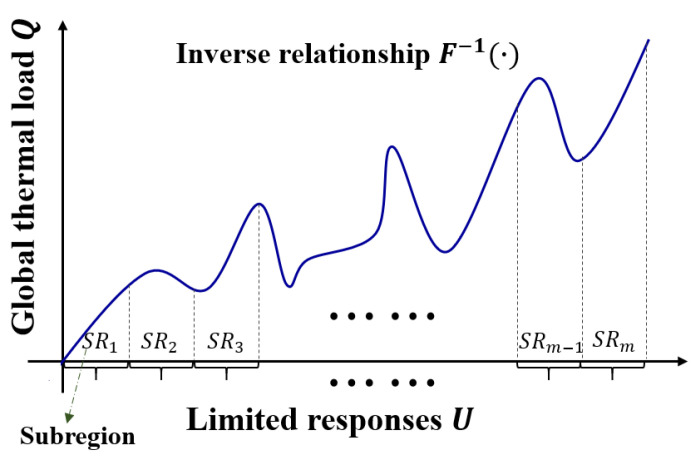
Complicated inverse load identification problem.

A complicated inverse relationship exists between the structural responses and thermal load, as shown in Figure 1, considering the complexity of industrial equipment and the discontinuity of thermal load positions throughout the entire structural domain, which is caused by the inaccessibility of heat sources under certain operating conditions. Moreover, heat sources only affect a small portion of large-scale industrial equipment. Sensors close to the load application region exhibit significant changes throughout the actual response measurement procedure, whereas sensors located farther from the load area detect slight or no change. Consequently, the local optima can be obtained by solving this problem using conventional direct identification techniques. In this study, the structure was partitioned into m subregions, and the challenging task of identifying thermal loads throughout the entire structure was segmented into a number of easier tasks to address load localization and magnitude evaluation across each subregion. The process is expressed as follows:

(1) $$label=f_{G}\left(U\right)$$

(2) $$x=f_{L1}\left(BC^{label},U^{label}\right)$$

(3) $$q=f_{L2}\left(x,BC^{label},U^{label}\right),$$

where label=1,2,⋯,m represents the subregion label, and $U^{label}\in {\mathbb{R}}^{p}$ is the vector of response data from p sensors in the subregion label. Furthermore, p⋅m≥n because of sensor sharing across nearby subregions. A *k*-dimensional vector, i.e., $BC^{label}\in {\mathbb{R}}^{k}$, represents the boundary information of the label-th subregion. The inclusion of boundary characteristics is supported by the fact that the subregions serve various functions, are positioned differently in the structure, and have distinct boundary characteristics during thermodynamic and mechanical inspections.

**Figure 2:**
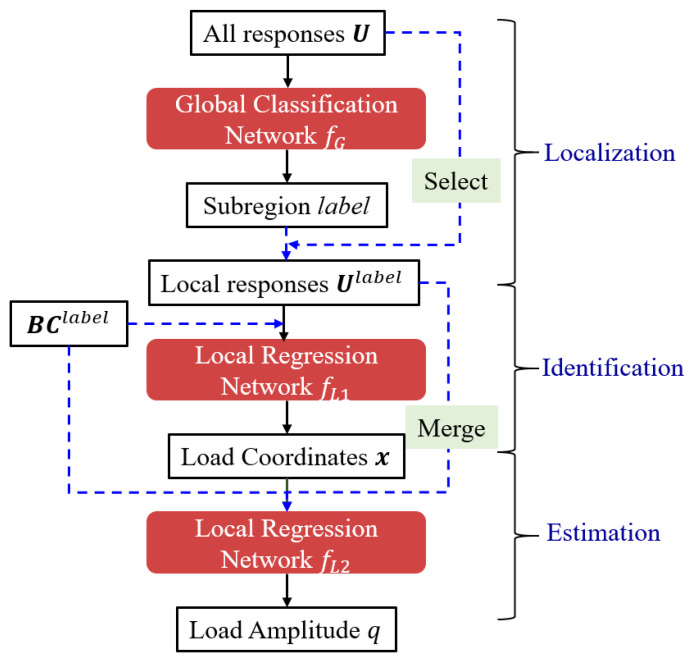
Flowchart of stepwise identification method.

Figure 2 shows the three steps of the structural thermal load identification process based on the stepwise identification method: localization, identification, and estimation. These modules are used to determine the subregion, position, and intensity of the load, respectively.

Step 1: Segment the structure into m geometrically similar subregions.

Step 2: To identify the subregion where thermal load is exerted, input the measured responses U from all the sensors into the global classification neural network $f_{G}$. The output is the corresponding subregion label.

Step 3: Select the appropriate measured responses $U^{label}$ of the subregion from U based on the label obtained in Step 2.

Step 4: Identify the thermal load position x; enter $U^{label}$ and the subregion boundary features $BC^{label}$ into the local regression network $f_{L1}$.

Step 5: Estimate the magnitude of thermal load q, input x, $BC^{label}$, and $U^{label}$ into the local regression network $f_{L2}$.

**Figure 3:**
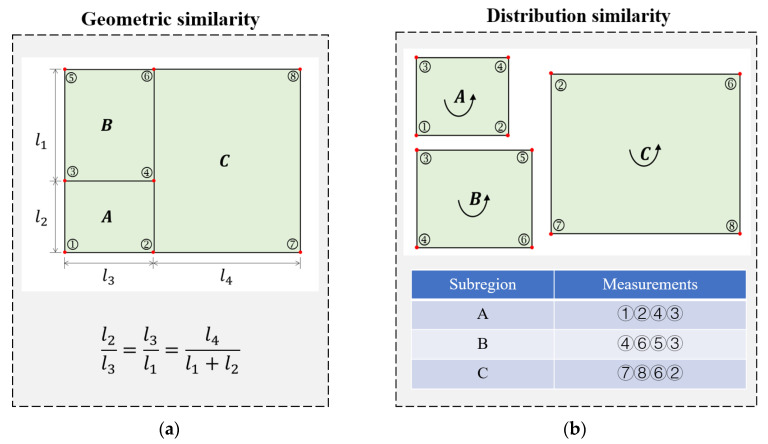
Principles of subregion segmentation and measurement point selection. (Letters A, B, and C represent the division of the structure into three subregions, and the numbers indicate the labels of the measurement points. For example, ① represents the first measurement point) (**a**) Subregions with geometric similarity. (**b**) Subregion measurement points with distribution similarity.

This method requires only three neural networks because the local regression networks $f_{L1}$ and $f_{L2}$ are shared by all subregions. In this case, the following aspects are noteworthy: (1) All subregions exhibit shape consistency, i.e., all subregions have the same shape and size ratio (Figure 3a). (2) The sensor layout of the subregions shows distribution consistency, which indicates that each subregion has the same number and placement of monitoring points (Figure 3b).

### 2.2. Model Architecture

**Figure 4:**
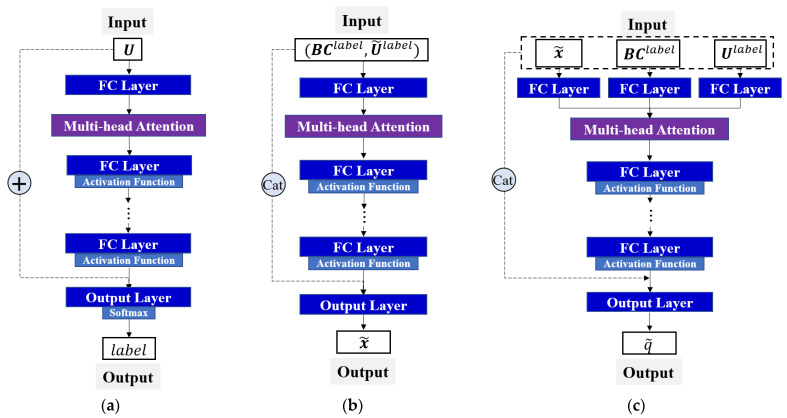
Three neural networks (refer to Figure 2) developed in this study (“+” and “Cat” represent addition and concatenation operations between two tensors, respectively). (**a**) Global classification network $f_{G}$; (**b**) local regression network $f_{L1}$; (**c**) local regression network $f_{L2}$.

In this study, three deep neural networks were developed to determine the thermal load parameters (see Figure 4), i.e., a global classification neural network $f_{G}$ (Figure 4a) and local regression neural networks $f_{L1}$ (Figure 4b) and $f_{L2}$ (Figure 4c).

$f_{G}$ locates the subregions of the thermal load exerted by sensing the change in data characteristics between measurements U and outputting the subregion label, in which label is an *m*-dimensional one-hot encoded vector. To enhance the feature extraction ability of the model for the input variables in the deep learning model, we introduce a multi-head attention layer, which is expressed as follows:

(4) $$label=\mathrm{Softmax}\left({\mathrm{FC}}_{\mathrm{out}}\left(\eta +U\right)\right)$$

(5) $$\eta =FC_{2:N_{G}}\left(\mathrm{Atten}\left({\mathrm{FC}}_{1}\left(U\right)\right)\right)$$

where Softmax(⋅) is the softmax activation function, Atten(⋅) is the multihead attention layer, FC(⋅) is the fully connected layer, $N_{G}$ is the number of hidden layers, and η is the latent feature of the $N_{G}$-th hidden layer. ${\mathrm{FC}}_{1}\left(\cdot \right)$ does not follow an activation function, and local regression neural networks $f_{L1}$ and $f_{L2}$ also perform this operation.

$f_{L1}$ is used to identify the thermal load position by inputting the boundary information $BC^{label}$ and normalized response ${\tilde{U}}^{label}$ of the subregion, as well as the output of the normalized coordinate $\tilde{x}$ of the thermal load. It can focus more on the relative changes between the structural responses and thus the structural response in this case is normalized. To unify the variation range of the load position, $\tilde{x}$ must be normalized because the subregion exhibits different variation ranges in different sections of the structure.

$f_{L2}$ quantitatively estimates the magnitude q of the thermal load by providing the thermal load coordinate $\tilde{x}$, boundary information $BC^{label}$, and response $U^{label}$. Because q is related to the absolute change trend of the monitoring response history and not the relative change between the responses of each sample, the original response data $U^{label}$ are utilized as the input variable. To achieve the best performance, the input variables must be converted based on the different task requirements.

## 3. Performance Evaluation

In this section, two examples are presented: the point heat source identification of a two-dimensional (2D) plate and the distributed heat load identification of a three-dimensional (3D) box structure. They were used to demonstrate the efficacy of the multi-neural network approach and stepwise identification method proposed herein in solving the inverse problem compared with a single neural network for identifying thermal loads. The most significant difference between the multi-neural network method and the stepwise identification method is that the former requires m+1 neural networks (i.e., each subregion corresponds to a regression neural network), while the latter only needs to build three neural networks (the $f_{L1}$ and $f_{L2}$ are shared on all subregions).

The global relative error (GRE) and coefficient of determination ($R^{2}$) were used to evaluate the performance of the neural network on the test dataset. The GRE and $R^{2}$ are defined as follows:

(6) $$GRE=\frac{\sum_{i=1}^{N}\left|y_{i}-{\tilde{y}}_{i}\right|}{\sum_{i=1}^{N}\left|y_{i}\right|}$$

(7) $$R^{2}=\frac{N\sum_{i=1}^{N}y_{i}{\tilde{y}}_{i}-\sum_{i=1}^{N}y_{i}\sum_{i=1}^{N}{\tilde{y}}_{i}}{\sqrt{\left(N\sum_{i=1}^{N}{\left(y_{i}\right)}^{2}-{\left(\sum_{i=1}^{N}y_{i}\right)}^{2}\right)\left(N\sum_{i=1}^{N}{\left({\tilde{y}}_{i}\right)}^{2}-{\left(\sum_{i=1}^{N}{\tilde{y}}_{i}\right)}^{2}\right)}}$$

where ${\tilde{y}}_{i}$ and $y_{i}$ represent the predicted values of the deep learning model and the target value, respectively, and N denotes the capacity of the dataset. The degree of difference between predictions and targets was determined based on $R^{2}$, which ranges from 0 to 1, where a value closer to 1 indicates a greater degree of correlation and a less significant difference between predictions and targets.

### 3.1. Example 1: Identification of a Point Heat Source on a 2D Plate

**Figure 5:**
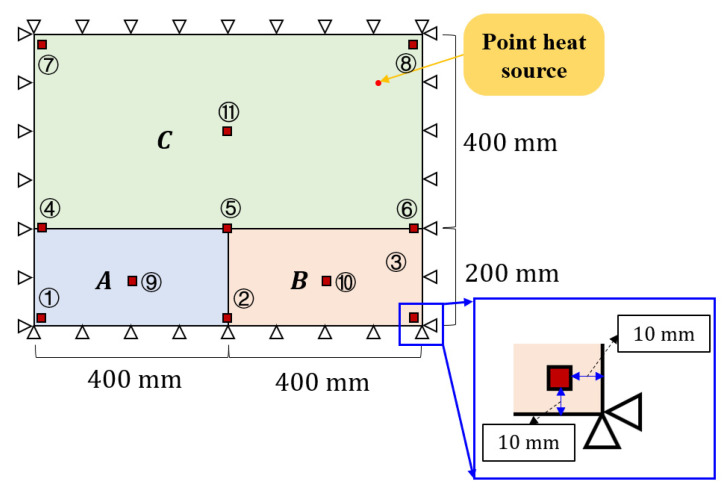
Plate with sensor layout. (Letters A, B, and C represent the division of the structure into three subregions, and the numbers indicate the labels of the measurement points. For example, ① represents the first measurement point).

To perform sample accumulation, finite element simulations are necessary. As shown in Figure 5, a point heat source was identified on a plate with four clamped edges. The structure measured 800 mm×600 mm×5 mm, and the material parameters used were as follows: elastic modulus $E=70\times 10^{3}$ MPa, Poisson’s ratio μ=0.33, coefficient of linear thermal expansion $\alpha =23.5\times 10^{-6}\;{}^{\circ}C^{-1}$, and thermal conductivity λ=0.237 W/(mm·K). 

Using the commercial finite element software ABAQUS v6.14.4, a thermal–mechanical coupled analysis of the structure was performed under an ambient temperature of 20 °C. To obtain the structural displacement response, 11 measurement points on the structure were selected, as shown in Figure 5, and their detailed placements are listed in Table 1.

**Figure 6:**
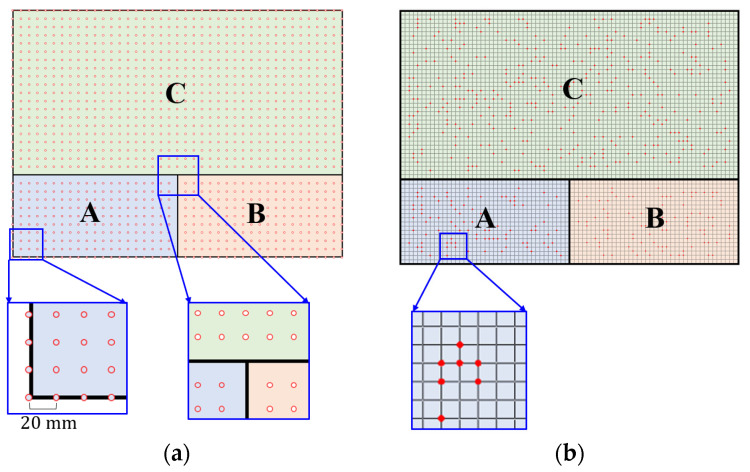
Sampling of training and test sets in example 1. (Letters represent labels for subregions; for example, A represents subregion A). (**a**) Training set. (**b**) Test set.

To demonstrate the completeness of the training dataset and the randomness of the testing dataset, two sampling methods were used, as shown in Figure 6. Equidistant sampling was employed for the training set, as shown in Figure 6a. The load position sampling interval was 20 mm, the load intensity sampling interval was 10 °C (from 50 °C to 150 °C). Sampling was not performed at the junctions of the subregions to prevent numerical errors in the global classification neural network $f_{G}$. In total, 12,969 training set samples were generated. The test set is shown schematically in Figure 6b. The point heat source generated 100, 100, and 400 random loading positions on subregions A, B, and C, respectively. A temperature (between 50 °C and 150 °C) was randomly selected as the thermal load magnitude at each loading position, which resulted in 600 samples in the test set.

The direct method of the classical single-neural network and the multi-neural network combination method were used to identify the thermal load. The direct method is required to establish a neural network. All displacement response data of the structure were used as the input, and the outputs were the position $\left(x_{1},x_{2}\right)$ and magnitude q of the load. The multi-neural network combination method can be categorized into two steps. First, a global classification neural network $f_{G}$ was used to locate the subregion; subsequently, a neural network associated with that subregion was activated to output the position $\left(x_{1},x_{2}\right)$ and magnitude q of the load. Therefore, four neural networks were established. The detailed neural network hyperparameters are listed in Appendix A Table A1.

**Figure 7:**
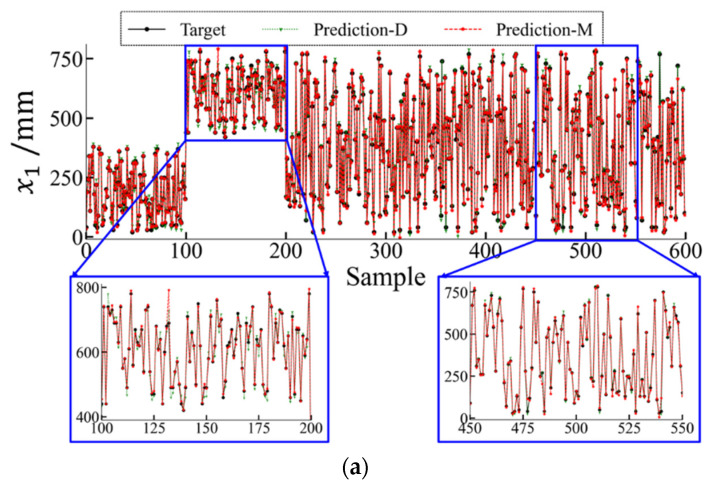

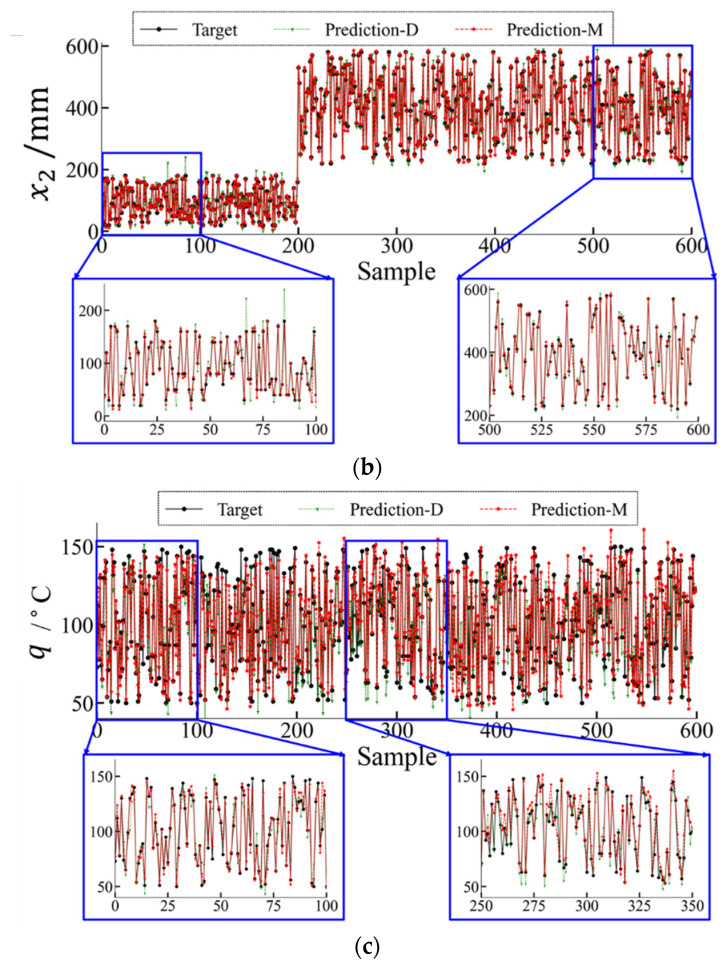
Comparison of thermal load identification between direct method (Prediction-D) and multi-neural network combination method (Prediction-M). (**a**) Thermal load coordinate $x_{1}$; (**b**) thermal load coordinate $x_{2}$; (**c**) thermal load amplitude q.

Table 2 presents the localization results of the classification neural network using a combination of multiple neural networks. The rows represent the predicted classes (the output of the deep learning model), whereas the columns represent the actual classes (the target output). The diagonal entries indicate the consistency between the predictions and targets. As shown, the accuracy reached 100%, which demonstrates the excellent performance of the established global classification neural network. Figure 7 presents the actual and predicted values of the load position and magnitude, where “Prediction-D” and “Prediction-M” represent the results of the direct method with a single neural network and the multi-neural network method, respectively. The load position and magnitude predicted by the multi-neural network method were more similar to the actual results compared with those predicted by the direct method.

**Table 2:** Prediction results of global classification neural network $f_{G}$ in example 1.

	Output A	Output B	Output C
Target A	100	-	-
Target B	-	100	-
Target C	-	-	400

The results of the error analysis for both methods are listed in Table 3. As shown, both methods indicated identification errors of less than 8%, with the identification errors for the load position being approximately 3%. This shows that the neural networks can model the relationship between local responses and the overall thermal load. The GRE decreased from 2.65% (Prediction-D) to 2.24% (Prediction-M), based on a comparison of the identification results for the load position coordinate $x_{1}$. The multi-neural network method increased the identification accuracy by 15.47% compared with the direct method. Additionally, the multi-neural network method increased the identification accuracy by 25.76% for the load position coordinate $x_{2}$ and by 15.72% for the magnitude q. Hence, a substantial advantage of the multi-neural network method using multiple neural networks over the use of a single neural network for load identification was demonstrated.

However, as shown in Table A1, the number of neural networks that must be established by a multi-neural network is the same as the number of subregions, which renders the application of this method to complex large-scale structures challenging. When applying deep learning, the training of neural networks is the most time-consuming and resource-intensive task. In addition, the identification errors of the load position and magnitude differ significantly. In this study, the magnitude of the identification error was 6.65%, whereas the position identification errors of the multiple neural networks were less than 3%. This is because the neural network cannot easily balance the two types of variables when they are used as outputs. Therefore, the multi-neural network combination method must be improved, which includes reducing the number of required neural networks and addressing the imbalance between the different output requirements. This is another motivation for this study.

### 3.2. Example 2: Distributed Thermal Load Identification for a Box Structure

**Figure 8:**
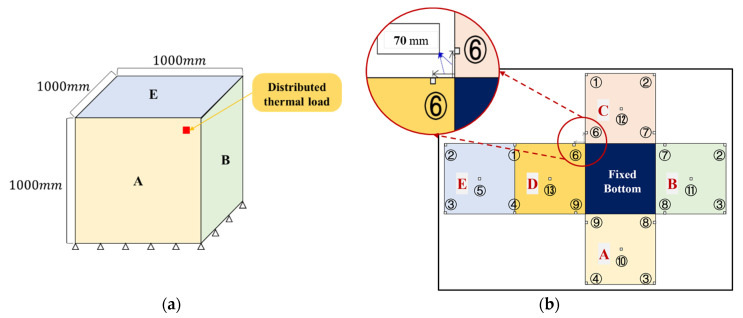
D structure and its subregions with sensor layout. (Letters A, B, C, D and E represent the division of the structure into five subregions, and the numbers indicate the labels of the measurement points. For example, ① represents the first measurement point) (**a**) 3D box-shaped structure. (**b**) Subregion expansion.

This section focuses on the identification of a uniformly distributed thermal load on the external surface of a box-type structure. The center position $\left(x_{1},x_{2},x_{3}\right)$ and intensity q of the uniformly distributed thermal load are parameters that need to be identified. As shown in Figure 8a, the structure comprises a top panel, a fixed boundary at the bottom, and four side panels. The four side panels and the top panel were the only panels used as the thermal load application area and for selecting displacement monitoring points. The structure measured 1000 mm × 1000 mm×1000 mm, and each side panel featured a thickness of 10 mm. A uniformly distributed heat load was applied over a 2 mm×2 mm area at a certain temperature. The material parameters of the structure were consistent with those described in Section 3.1. The environmental temperature was set to 20 °C, and a steady-state thermal-structural finite element analysis was performed to generate samples.

**Figure 9:**
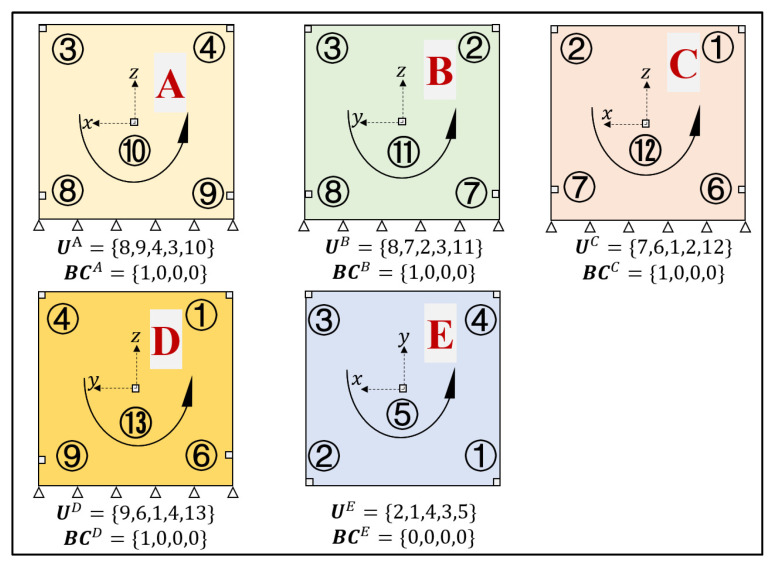
Boundary conditions and measurement point placement of five subregions. (Letters A, B, C, D and E represent the division of the structure into five subregions, and the numbers indicate the labels of the measurement points. For example, ① represents the first measurement point).

Monitoring points were specified on the four side panels and the top panel, as shown in Figure 8b. A total of 13 displacement measurement points were selected, with the four points near the bottom set at a distance of 70 mm from the bottom. The other nine measurement points are placed at the upper four corners, midpoints of the four sides, and the midpoint of the top surface of the structure. The detailed monitoring point layout for each subregion is shown in Figure 9. The encoding principle for the BCs is as follows: Beginning from the bottom side and moving anticlockwise, each of the four sides of the subregion correspond to a component of the encoding vector. The others are encoded as 0, whereas the fixed boundary is encoded as 1.

**Figure 10:**
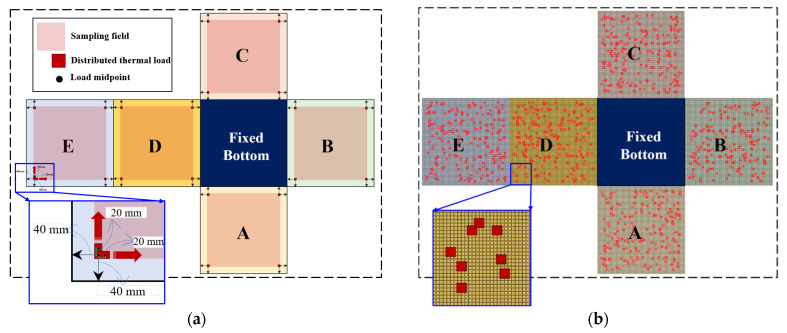
Sampling of training and test sets in example 2. (Letters A, B, C, D and E represent the division of the structure into five subregions) (**a**) Training set. (**b**) Test set.

As shown in Figure 10, two sampling methods, i.e., equidistant and random sampling, were used to accumulate the training and testing samples. The sampling area for the thermal load was set 40 mm from the boundary of the subregion. As shown in Figure 10a, the sampling interval of the training set for the heat load position was 20 mm, and the magnitude was sampled at intervals of 20 °C between 300 °C and 400 °C. Therefore, the training set contained 28,830 samples. As for the test set, in each subregion’s loading area, 200 thermal load loading positions were randomly selected (Figure 10b), and one value for the load magnitude was selected between 300 °C and 400 °C. The test set contained 1000 samples.

The results of using the global classification neural network $f_{G}$ for thermal load localization in the stepwise method are presented in Table 4. Based on the findings, the established model achieved an accuracy of 99.8%. However, after examining the predicted results, we discovered that the remaining 0.2% was caused by two samples whose thermal load was in close proximity to subregions C and E at relatively low heat load magnitudes. The displacement responses at the measurement points on these two subregions exhibited similar fluctuation patterns.

**Table 4:** Prediction results of global classification neural network $f_{G}$ in example 2.

	Output A	Output B	Output C	Output D	Output E
Target A	200	-	-	-	-
Target B	-	200	-	-	-
Target C	-	-	200	-	-
Target D	-	-	-	200	-
Target E	-	-	2	-	198

**Figure A1:**
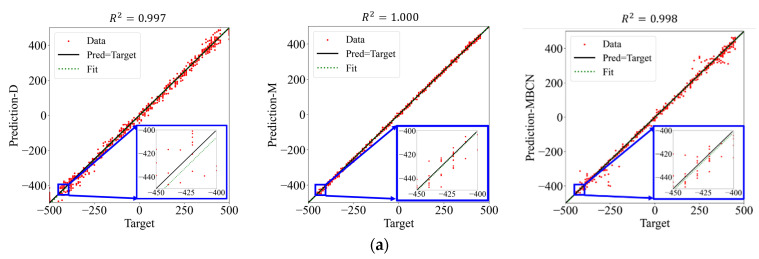

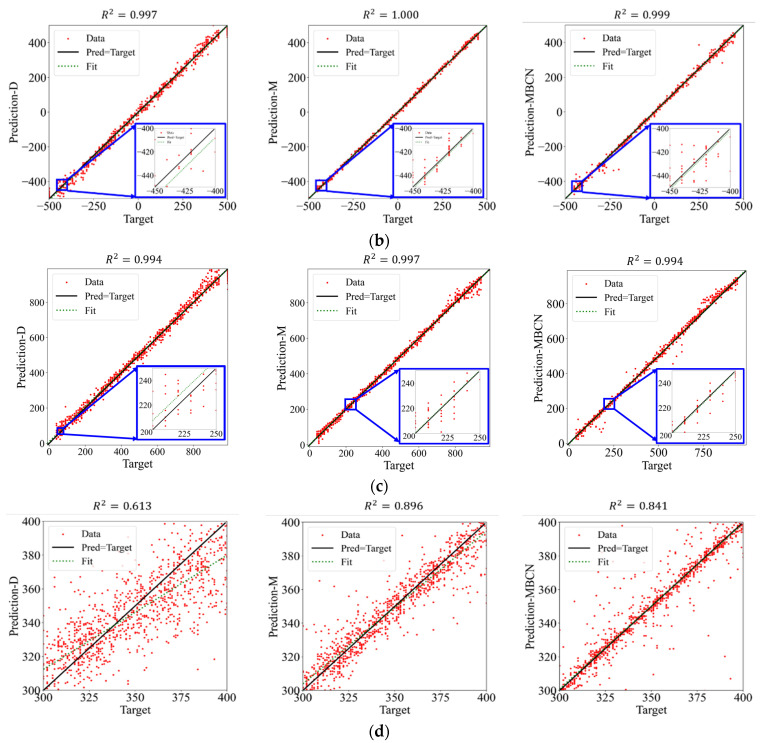
Correlation analysis between the predictions and the target values. (**a**) Thermal load coordinate $x_{1}$. (**b**) Thermal load coordinate $x_{2}$. (**c**) Thermal load coordinate $x_{3}$. (**d**) Thermal load magnitude q.

Table 5 presents the recognition results of the uniform thermal load using the direct, multi-neural network combination, and stepwise identification methods. Detailed regression analysis plots are shown in Figure A1 in the Appendix A. Here, “Prediction-MBCN” is used as the stepwise identification method because it incorporates multiple neural network modules, BC encoding modules, cascaded neural network modules for heat load position and intensity estimation, and noise injection training for the intensity estimation network. As shown in Figure 4c, noise was introduced to enhance the robustness of the deep learning model during the estimation of the thermal load magnitude. This is because, to estimate the magnitude of the thermal load, the output variable of $f_{L1}$ was used as the input variable of $f_{L2}$, which necessitates the introduction of 0.5% Gaussian noise into $\tilde{x}$ during the training of $f_{L2}$.

Table A2 in the Appendix A lists the specific hyperparameters of the neural network. Table A2 shows that, in contrast to the multi-neural network method, the local regression neural network $f_{L1}$ of the stepwise identification method requires only two output variables to identify the thermal load on the subregions. This is because, when analyzing the external load identification problem of a box structure, partitioning the structure into smaller segments allows the 3D overall thermal load localization problem to be transformed into a 2D subregion problem (Figure 8), thereby necessitating the identification of only two coordinates of the load position. All the subregions shared the local regression neural networks $f_{L1}$ and $f_{L2}$ after the load parameters were normalized, as compared with the result of the multi-neural network method. Therefore, the construction and training costs of the deep learning model decreased significantly. In particular, the stepwise identification method significantly reduced the learning rate and training epochs during the training of deep learning models, as well as the number of deep learning models required.

As shown in Table 5, the stepwise identification method and multi-neural network method achieved similar identification accuracies, which were significantly higher than that of the direct method. This confirms the effectiveness of using multiple neural networks to address complex problems. The stepwise identification method significantly outperformed the direct method in terms of both the GRE and $R^{2}$. The thermal load parameters $x_{1}$, $x_{2}$, $x_{3}$, and q reduced by 60.99%, 68.61%, 45.36%, and 58.65%, respectively. However, compared with the multi-neural network approach, in which each subregion has its own neural network, the stepwise identification method utilizes fewer shareable neural networks for thermal load identification. Although a slight decrease in accuracy is indicated, this method significantly reduces the model construction and training time, thereby improving the prediction efficiency. Additionally, the identification errors for the load position and magnitude based on the stepwise identification method were within 1.5% and 2%, respectively, which indicates insignificant differences among them. In conclusion, the proposed stepwise identification method achieved high-precision identification of distributed thermal loads on the box structure. It can also solve numerous neural network training problems via the multi-neural network method and ensure that the prediction accuracy of different types of output variables under the same framework is similar.

## 4. Ablation Analysis

**Figure 11:**
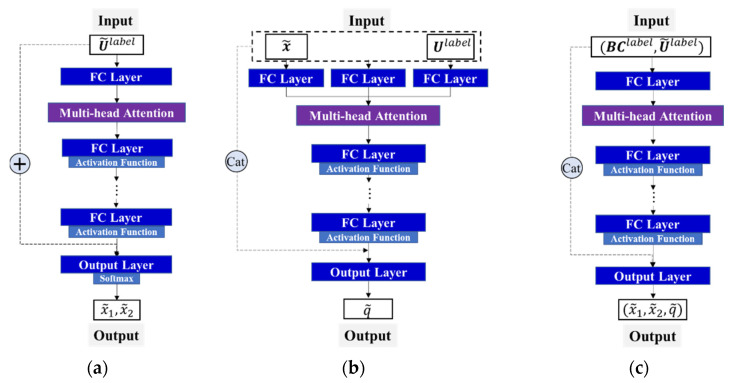
Deep learning model architecture with removed modules. (**a**) Local regression network $f_{L1}$ without BCs. (**b**) Local regression network $f_{L2}$ without BCs. (**c**) Local regression network $f_{L}$ without cascading.

To investigate the specific performance of several components of the proposed method, an ablation experiment was performed. Each component was removed from the stepwise identification method (Prediction-MBCN), as shown in Figure 11, and its performance was compared with that of the original architecture. The hyperparameters of the neural networks presented in this section are listed in Table A3.

Multiple neural networks: As shown in the previous section, the accuracy of the multiple neural networks in identifying thermal loads is significantly higher than that of direct prediction via a single neural network (Prediction-D); therefore, it will not be addressed in detail in this section.

Coding of BCs: As shown in Figure 11a,b, ablation was performed by removing the BC data from the input variables of the local regression network.

Cascaded neural network: By combining the outputs of local regression networks $f_{L1}$ and $f_{L2}$, a local regression neural network $f_{L}$ was designed to directly output the load position and magnitude (refer to Figure 11c).

Noise introduced into network training: The structural heat load position from the training set was used directly as the network input without any noise perturbation during the training of the magnitude-estimation neural network (Figure 4c).

**Figure 12:**
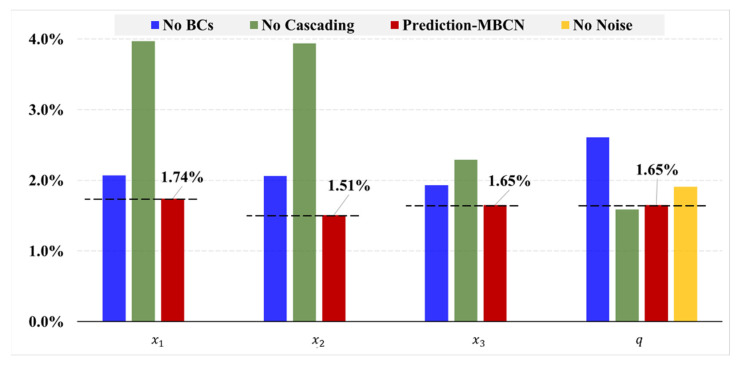
Changes in GRE across ablation tests.

Figure 12 shows that the original architecture of the stepwise identification method outperformed other deep learning models with removed components in most of the thermal load parameter identification tasks. In particular, the errors were between 1.5% and 2%, which were similar to those generated when the cascaded neural network was used to separately estimate the position and magnitude of the thermal load. However, when a non-cascaded neural network (Figure 11c) was used, the identification error of the load position was much larger than that of the load magnitude.

To quantitatively analyze whether the ablation experiments affected the proposed deep learning model, the GRE for each neural network is shown in Table 6. The identifying errors for load positions $x_{1},\;x_{2},\mathrm{and}\;x_{3}$ and magnitude q increased by 18.97%, 36.42%, 16.97%, and 58.18%, respectively, without the BCs. The primary reason for this difference is that different deformation patterns exist when the boundary conditions of subregions differ. In other words, even if two substructures have the same thermal load position and magnitude, the observed responses may differ, and vice versa. As a result, even though the relevant input variables are the same, the output variables are not unique. Therefore, the absence of boundary conditions would lead to an increase in identification errors for thermal loads. When the cascaded neural network was not used, the recognition errors for thermal load positions $x_{1},x_{2}$, and $x_{3}$ increased by 128.16%, 160.93%, and 38.79%, respectively, whereas the estimation error for the thermal load magnitude q decreased slightly. This is because developing a cascaded neural network to account for the coupling output of two different variables inevitably causes the prediction errors to propagate from one neural network to the next. However, using the cascaded neural network significantly increased the position identification accuracy while only marginally deteriorating the performance of the magnitude estimation by 3.64%. Similarly, the recognition error decreased by 15.76% when Gaussian noise was introduced to the input variables of the load positions during the deep learning model training for thermal magnitude estimation. The reason for this is that when $f_{L2}$ performs thermal load magnitude estimation, its input variables include the predicted values of thermal load positions (refer to Figure 2). Introducing Gaussian noise is used to simulate the prediction error of the thermal load positions of $f_{L1}$, significantly enhancing the robustness of $f_{L2}$. In general, all the components enhanced the overall performance of the model and achieved high accuracies in thermal load identification.

## 5. Conclusions and Outlooks

A stepwise identification method for the high-precision identification of structural thermal loads, including their location and magnitude, was proposed herein. By combining several deep learning models and examining the potential correlation between a few structural responses and the overall load, the method derived from a data-driven paradigm solved the load identification inverse problem. Two examples were presented to evaluate the proposed method, i.e., the identification of a point thermal source for a 2D plate and the identification of a uniformly distributed thermal load for a 3D box-shaped structure. The main conclusions obtained were as follows:

The stepwise identification method significantly improved the identification accuracy of structural thermal loads, including their positions and magnitudes. Compared with direct methods for a single deep learning model, the stepwise identification method improved the accuracy by more than 45%.

The introduction of a cascaded neural network reduces imbalances in prediction errors among multiple types of output variables. In the thermal load identification of a 3D box-type structure, the identification errors for thermal load positions and intensity parameters using the stepwise identification method are both around 1.6%.

The generalization ability of the algorithm can be improved by encoding the boundary information of the subregion into a deep learning model. This allows the subregions in different sections of the overall structure to be considered and the use of different connection functions. In the ablation analysis section, it was observed that the identification errors for thermal load positions and magnitude parameters decreased by approximately 15.94%, 26.70%, 14.51%, and 36.78%.

However, there are still areas that provide opportunities for further exploration, such as the research on thermal load identification for structures with complex geometric shapes and practical engineering application issues of the proposed method. Some preliminary insights are as follows:

Due to the current method being applicable only to spacecraft structures that can be divided into multiple geometrically similar subregions, there is a need for improvement in the subregion subdivision approach. It is imperative to propose a subregion mapping method suitable for complex geometric shapes. This method should enable the subdivision of structures with any complex shape into several regular subregions.

The current neural network model relies on finite element simulation data, which ensures high data purity. Therefore, it is essential to conduct thermal load tests on real satellite structures and establish a dataset based on experimental data. To strengthen the robustness of the thermal load identification model and apply it in real engineering, this step is essential.

## Figures and Tables

**Table 1:** Sensor layout of panel subregions.

Subregion	Measurements
A	①②⑤⑥⑨
B	②③⑥⑤⑩
C	④⑥⑧⑦⑪

**Table 3:** GRE comparison of direct method (Prediction-D) and multi-neural network combination method (Prediction-M).

GRE	$x_{1}$	$x_{2}$	q
Prediction-D	2.65%	3.30%	7.89%
Prediction-M	2.24%	2.45%	6.65%
Improvement	15.47%	25.76%	15.72%

**Table 5:** Thermal load identification accuracy of direct method (Prediction-D), multi-neural network combination method (Prediction-M), and stepwise identification method (Prediction-MBCN).

	GRE				$R^{2}$			
	$x_{1}$	$x_{2}$	$x_{3}$	q	$x_{1}$	$x_{2}$	$x_{3}$	q
Prediction-D	4.46%	4.81%	3.02%	3.99%	0.997	0.997	0.994	0.613
Prediction-M	1.14%	1.58%	1.58%	1.84%	1.000	1.000	0.997	0.896
Prediction-MBCN	1.74%	1.51%	1.65%	1.65%	0.998	0.999	0.994	0.841

**Table 6:** Thermal load identification error after component removal in ablation tests.

	$x_{1}$	$x_{2}$	$x_{3}$	q
No BCs	2.07%	2.06%	1.93%	2.61%
No cascading	3.97%	3.94%	2.29%	1.59%
No noise	-	-	-	1.91%
Prediction-MBCN	1.74%	1.51%	1.65%	1.65%

## Data Availability

Data are contained within the article.

## Appendix A

**Table A1:** The hyperparameters of the neural networks in Section 3.1.

Hyperparameters		Structure Hyperparameters					Training Hyperparameters		
		Activation Function	Hidden Layers	Input Size	Output Size	Head Number	Batch Size	Learning Rate	Epochs
Direct method	-	Sigmoid	(8, 15, 20, 10)	11	3	-	128	0.01	2500
Multiple networks combined	$f_{G}$	Sigmoid	(8, 16, 20, 11)	11	3	8	128	0.001	2500
	$f_{LA}$	Sigmoid	(8, 15, 26, 10)	5	3	-	128	0.01	2500
	$f_{LB}$		(8, 15, 25, 10)						3000
	$f_{LC}$		(8, 15, 30, 10)						2500

**Table A2:** The hyperparameters of the neural networks in Section 3.2.

Hyperparameters		Structure Hyperparameters					Training Hyperparameters		
		Activation Function	Hidden Layers	Input Size	Output Size	Head Number	Batch Size	Learning Rate	Epochs
Direct method	-	ReLU	(15, 20, 10]	13	4	-	128	0.0005	5000
Multi-neural networks method	$f_{G}$	Sigmoid	(16, 20, 11]	13	5	8	256	0.001	3300
	$f_{LA}$	LeakyReLU	(15, 30, 25, 10)	5	3	-	64	0.003	8000
	$f_{LB}$		(15, 30, 25, 10)					0.0005	10,000
	$f_{LC}$		(15, 30, 20, 10)					0.003	8000
	$f_{LD}$		(15, 30, 25, 10)					0.003	8000
	$f_{LE}$		(15, 30, 25, 10)					0.003	9000
Stepwise identification method	$f_{G}$	Sigmoid	(16, 20, 11)	13	5	8	256	0.001	3300
	$f_{L1}$	ReLU	(16, 30, 25, 10)	5 + 4	2	8	128	0.002	2000
	$f_{L2}$	ReLU	(16, 30, 25, 10)	5 + 2 + 4	1	8	128	0.001	4000

**Table A3:** The hyperparameters of the neural networks in Section 4.

Hyperparameters		Structure Hyperparameters					Training Hyperparameters		
		Activation Function	Hidden Layers	Input Size	Output Size	Head Number	Batch Size	Learning Rate	Epochs
No BCs	$f_{L1}$	ReLU	(16, 30, 25, 10)	5	2	8	128	0.002	2000
	$f_{L2}$	LeakyReLU	(16, 30, 25, 10)	5 + 2	1	8	128	0.0001	4000
No cascading	$f_{L}$	ReLU	(16, 30, 25, 10)	5 + 4	3	8	128	0.002	2000
No noise	$f_{L2}$	ReLU	(16, 30, 25, 10)	5 + 4 + 2	1	8	128	0.001	4000
